# Understanding hearing health‐care access in Australia: Users' perspectives

**DOI:** 10.1111/ajag.70029

**Published:** 2025-04-22

**Authors:** Shermin Lim, Jessica Turner, Diana Tang, Kerry Sherman, Kompal Sinha, Sharad Chawla, Simon Carney, Giriraj Singh Shekhawat, Bamini Gopinath

**Affiliations:** ^1^ College of Education, Psychology & Social Work Flinders University Adelaide South Australia Australia; ^2^ Macquarie University Hearing, Department of Health Sciences Macquarie University Sydney New South Wales Australia; ^3^ School of Psychological Sciences Macquarie University Sydney New South Wales Australia; ^4^ Department of Economics Macquarie Business School Sydney New South Wales Australia; ^5^ Department of Otorhinolaryngology Flinders Medical Centre Adelaide South Australia Australia; ^6^ College of Medicine and Public Health Flinders University Adelaide South Australia Australia; ^7^ Tinnitus Research Initiative Regensburg Germany

**Keywords:** adult, cochlear implants, hearing aids, hearing loss, self‐management

## Abstract

**Objective:**

The aim of this qualitative study was to evaluate the barriers and enablers to current hearing health‐care services in Australia for middle‐aged and older adults who use cochlear implants (CI) and/or hearing aids.

**Methods:**

Adults aged 40 years and older from the Hearing impairment Adults: a Longitudinal Outcomes Study (HALOS), with adequate English language skills, were invited to participate in a semi‐structured interview about their hearing intervention journey. A thematic analysis was applied to the interview transcripts via inductive coding.

**Results:**

Thirty‐one hearing device users (15 hearing aid users, 9 CI users and 7 bimodal users) across Australia enrolled in the interview. Themes identified include hearing care management, alternative support services, patient self‐management and accessibility to hearing services.

**Conclusions:**

Findings indicated the need for primary health and hearing care professionals to reconsider their clinical approach with individuals with hearing loss. Rehabilitation, primary health and hearing care services ought to work together to create an integrated hearing care journey for patients.

**Policy impact**

This study indicates that non‐government agencies, primary health and hearing care services should work together to create an integrated hearing care journey. Addressing these aspects through a multi‐faceted approach is essential for establishing equitable and efficient hearing health‐care systems, ultimately enhancing the quality of life for adults with hearing impairment.

## 1 INTRODUCTION

Hearing impairment is highly prevalent in Australia.^1^, ^2^ One in 10 (12%) adults aged 65 years and older has a hearing impairment (partial or complete deafness), with a higher proportion of males affected (40%) than females (25%).^1^ In the 30–59 years age group, one in 20 is affected among those aged 45–59 years, a higher prevalence than in those aged 30–44 years.^2^ Despite being a common condition, adult‐onset hearing loss remains under‐recognised and is frequently referred to as an ‘invisible disability’.^3^, ^4^

Hearing impairment has a significant impact on both individuals and society.^4^ For individuals, there is a significant impact on communication, leading to psychological distress,^3^ reduced quality of life^4^ and decreased social connectedness.^3^ Economic impacts include increased welfare payments and health‐care costs linked to hearing loss.^5^

Numerous studies have explored stages of the hearing health‐care pathway, including the role of primary health care in hearing management,^6^ hearing evaluation,^7^ hearing device management, such as hearing aid (HA) usage,^8^ and the postoperative experiences of cochlear implant (CI) users.^9^

To identify gaps in the hearing health‐care pathway, it is essential to examine the experiences and needs of the target population. Prior studies have highlighted challenges faced by individual groups, such as HA user^7^, ^8^ or CI users separately.^10^, ^11^ Few studies have conducted direct comparisons of the experiences and needs of these two groups in Australia, nor included device users aged 40 years and older; that is, examined the perspectives of device users from midlife through to older age. This underscores the novelty and significance of our study, as it provides valuable insights within an Australian context.

By comparing the two hearing device users, we can uncover unique insights and disparities in their access to and utilisation of hearing health‐care services. The comparison can reveal specific areas where one group may benefit more effectively than the other, helping to pinpoint opportunities for improvement. Addressing these gaps through a comparative approach can lead to a more efficient and equitable hearing health‐care system, ensuring that all individuals receive optimal care and support.

To address this knowledge‐gap, we analysed qualitative data collected from HA and/or CI users aged 40 years and older across Australia as part of the Hearing Impairment in Adults: A Longitudinal Outcomes Study (HALOS).^12^ The aim was to identify the differences between HA and CI users' needs and experiences in their hearing health‐care journey.

## 2 METHODS

This study was approved by the Macquarie University Human Research Ethics Committee (ID: 11262) and Southern Adelaide Clinical Human Research Ethics Committee (SAC HREC) (ID: LNR/22/SAC/88).

### 2.1 Participants and recruitment

Participants for HALOS were recruited from independent clinics, hospitals or large hearing chain providers across multiple states in Australia and were contacted by mail, social advertising or referral from their hearing care provider. The inclusion criterion for HALOS was aged 40 years or older, using HAs and/or CIs in at least one ear, and adequate English proficiency for communication in the interview. A purposive sampling strategy was used as we wanted to ensure equal numbers of HA and CI users.

### 2.2 Data collection

The semi‐structured interview guide was developed by the HALOS research team. Collaboratively, the researchers have expertise in fields, including audiology, public health and qualitative research methods.^12^ Interviews were conducted by two researchers via phone call or in person between October 2022 and July 2023. One researcher (JT) was the study coordinator with a background in health sciences, and the other (SL) was a PhD student with a background in audiology. There were eight open‐ended questions in the interview guide, which allowed participants to share their experiences of navigating the hearing health‐care pathway, such as their preferences and concerns about the treatment and management process for hearing intervention and devices provided (Appendix S1). Participants were asked to provide suggestions as to what aspects of the hearing care pathway could be improved or modified. All interviews were audio‐recorded with consent.

### 2.3 Data analysis

Audio recordings of the interviews were transcribed verbatim via Microsoft Word. Transcripts were imported into NVivo software and analysed using inductive thematic analysis,^13^ following the framework outlined by Braun and Clarke,^13^ to examine the participants' experience in a rich and cohesive manner. Two interviewers independently reviewed the transcripts and generated codes before meeting to discuss and achieve consensus on the themes and illustrative quotes. Discrepancies in coding were resolved through discussion with a third researcher (DT) (nutrition, public health).

Using text analysis via inductive coding, different key themes were extracted and categorised. These codes were integrated to determine the context, barriers to, and enablers of hearing intervention phases. Participants were recruited until data saturation was reached, where no new themes were identified.

## 3 RESULTS

### 3.1 Participant characteristics

Thirty‐one hearing device users (14 males, 17 females, mean age = 68.26 years, SD = 11.63) were recruited across seven Australian states. Participant demographics and hearing status collected through the HALOS online questionnaire are presented in Table 1. The average duration of the interviews was 27 min, ranging between 10 and 60 min.

**TABLE 1:** Characteristics of participants included in the interview at baseline.

ID	Gender	Age	Employment status	Side of hearing loss	Years of hearing loss	Cause of hearing loss	Current hearing device	Years of use HA	Years since implantation	Previous HA use prior to CI
CI01	Female	56.9	Employed	Both	>10	Disease, medications/chemicals	Bilateral CI	n/a	1st: 5–10 2nd: 5–10	Yes
CI02	Female	64.3	Retired	Both	>10	Not known	Unilateral CI	n/a	>10	Yes
CI03	Female	71	Retired	Both	>10	Disease	Bilateral CI	n/a	1st: >10 2nd: 5–10	Yes
CI04	Female	41.4	Employed	Both	>10	Disease, noise exposure	Bimodal	<1	>1	Yes
CI05	Male	47.6	Employed	Both	From birth	Medications/chemical	Bimodal	>10	1–5	Yes
CI06	Male	77.1	Pension	Both	>10	Noise exposure	Bimodal	>10	1–5	Yes
CI07	Male	54	Employed	Both	From birth	Unknown	Bilateral CI	n/a	1st: >10 2nd: 5–10	Yes
CI08	Male	78.2	Retired	Both	>10	Age‐related, disease, noise exposure	Bimodal	6–10	1–5	No
CI09	Female	48.3	Employed	Both	>10	Unknown	Bilateral CI	n/a	1st: >10 2nd: >10	Yes
CI10	Female	74.8	Pension	Both	>10	Hereditary	Unilateral CI	n/a	>10	Yes
CI11	Female	75.6	Employed	Both	>10	Unknown	Bilateral CI	n/a	1st: >10 2nd: >10	Yes
CI12	Male	69.6	Retired	Both	>10	Unknown	Unilateral CI	n/a	<1	No
CI13	Male	62.9	Employed	One	5–10	Accident	Unilateral CI	n/a	5–10	No
CI14	Male	75.1	Retired	Both	>10	Age‐related, noise exposure	Bimodal	>10	1–5	Yes
CI15	Female	60.1	Medical disability	Both	>10	Osteogenesis imperfecta	Bimodal	>10	1–5	Yes
HA01	Female	67.2	Retired	Both	>10	Unknown	Bilateral HAs	>10	n/a	n/a
HA02	Female	73.3	Retired	Both	>10	Disease	Bilateral HAs	>10	n/a	n/a
HA03	Male	77.9	Retired	Both	>10	Noise exposure	Bilateral HAs	1–5	n/a	n/a
HA04	Female	57.3	Employed	Both	From birth	From birth—rubella	Unilateral HA	>10	n/a	n/a
HA05	Female	66.5	Retired	Both	5–10	Age‐related, noise exposure	Bilateral HAs	1–5	n/a	n/a
HA06	Male	70.8	Pension	Both	>10	Medications/chemical	Bilateral HAs	6–10	n/a	n/a
HA07	Female	84.4	Retired	Both	5–10	Age‐related	Bilateral HAs	6–10	n/a	n/a
HA08	Male	77	Retired	Both	5–10	Age‐related	Bilateral HAs	6–10	n/a	n/a
HA09	Female	79.3	Retired	Both	>10	Unknown	Bilateral HAs	1–5	n/a	n/a
HA10	Female	67.9	Retired	Both	>10	Meniere's disease	Bilateral HAs	1–5	n/a	n/a
HA11	Female	85.1	Retired	Both	Unknown	Unknown	Bilateral HAs	>10	n/a	n/a
HA12	Male	82.7	Pension	Both	5–10	Age‐related	Bilateral HAs	6–10	n/a	n/a
HA13	Male	49.7	Employed	Both	>10	Noise exposure	Bilateral HAs	<1	n/a	n/a
HA14	Female	72.5	Retired	Both	5–10	Age‐related	Bilateral HAs	1–5	n/a	n/a
HA15	Male	67.2	Retired	Both	5–10	Noise exposure	Bilateral HAs	6–10	n/a	n/a

*Note*: 1st, first implantation; 2nd, time period of second implantation prior to years after first implantation.

Abbreviation: n/a, not applicable.

### 3.2 Themes

Four main themes surrounding middle‐aged and older adult access to hearing health care emerged: (i) hearing health‐care services, (ii) alternative support services, (iii) patient self‐management and (iv) accessibility to hearing services. Representative quotes for the themes are presented in Table 2.

**TABLE 2:** Representation quotes compared between hearing aid and cochlear implant users when accessing the healthcare services for their hearing loss.

Themes	Representative quote		Barrier/enabler
	Hearing aid	Cochlear implant	
**Theme 1: Hearing care management**			
(1) Primary health‐care services and specialists	‘They just referred me to an audiologist without explaining anything to do with how audiologists work in Australia’. (HA10) ‘My GP wasn't overly interested really, to be quite honest’. (HA01)	‘You talk about support from the GP, but there wasn't so much from the GP’. (CI07)	Barrier
	‘The next time I was at the GP I just mentioned it. You know I think I'm getting old. I'm losing my hearing and he said, oh, you've gotta have it all checked out… he referred me to an ENT guy’. (HA07)	‘When the hearing loss went suddenly last year like the GP, basically rang all the local audiologists and ENTs to get me in an emergency appointment and like advice on what I should do’. (CI05) ‘He explained, like how whilst we didn't know what caused this latest hearing loss, he's like there's probably like a flow on effect that it's all connected somehow and he just was really calm. Asked me if I had any questions. Asked my husband if he had any questions. Gave me like a brochure about like explaining hearing loss to kids so that you know I had something to come and you know, be able to explain it to my kids, what was gonna happen and, and that sort of thing’. (CI05)	Enabler
(2) Hearing services	‘From the minute I walked in the door, I felt comfortable with what was told to me, how I was treated, I was listened to and that was seriously important to me… Poor audiologist most probably needs a medal because I'm forever asking questions. But he's very generous with his time’. (HA09) ‘I've come across some really, really lovely people that have listened and advised and and that advice has been sound and I don't feel like I've been dismissed. I feel that I've been listened to and cared for’. (HA05)	‘They did another test and they said no, there was nothing else. There's nothing stronger, they could do nothing more, and they recommended they sent a letter to my doctor to advise me to go to [cochlear implant clinic] for a test’. (CI04) ‘I was very satisfied with the consultation with him, he was quite thorough. Warned me about some of the potential side effects… So, I found that the discussions on that quite educational to me and help me understand what was happening and how these things actually work… I'm more comfortable once I know how something works than just assuming it's going to’. (CI15) ‘Yep, they provide me with regular appointments. They've accommodated me around what I was working, when I was working and if I have any problems, I was having a bit of a problem I said something is not right. So, they booked me in for an earlier appointment’. (CI02)	Enabler
	‘There might be a couple of adjustments. But there's no aftercare in terms of well these are some of the strategies you use to make the whole hearing experience better. My view is, and I'm no expert, but I think the technology is half of the solution and the other half is using strategies to go with that. And you get a better experience’. (HA02) ‘I think it would be really good to, in that first appointment with a new customer… to really do an assessment of where they're at and why they're there and what changes that they want to make, so they can really gauge whether that person is ready to accepted hearing devices or whether they need to maybe come back in six months or something like that to think about it’. (HA02) ‘I do ask a lot of questions. I'm very inquisitive. I asked why are they doing this, that or the other? Information has always been freely given, so I feel fully informed the whole time. And sometimes that's a difficult process. I've generally got to probe at this heavily. But I haven't had to do that [at current hearing service provider]. I've asked a question; it's been openly answered and given to me. I don't think anyone can ask for anything better than that’. (HA09)		Both

‘They had a specific brand that to me they appeared to be pushing, but I wasn't happy with. I ended up stopped going there…’ (HA09) ‘…it's my hearing…I do ask a lot of questions. But I'd like to think I ask them politely. And I was fobbed off… And I can't see that answering a question should be a problem’. (HA09) ‘But it was just that you saw different people all the time. It was so nice when I was able to build up a relationship with somebody and she has been there each time that I went’. (HA11)	‘I think the biggest problem for the audiologists in the clinic, is that they actually don't have the time. They are not trained to think about the emotional, psychosocial consequences. And there needs to be a continuity of care… They never suggested any sort of rehabilitation and music is definitely ignored’. (CI03)	Barrier
**Theme 2: Alternative support systems and resources**			
(1) Social support services	‘I should add that my wife is pretty proactive about it and castigates me if I don't put my hearing aids in’. (HA08) ‘Being part of [Hearing Support Organisation], it's made me more confident or more assertive with it in terms of saying, well OK. This is what I need. This is what we both need to do so that we can communicate better’. (HA02)	‘It was very digital and exceedingly difficult… my wife persevered with me. We read every Doctor Seuss book there is. Or she read it, and I repeated it. … we did that every day for three months. And that that got me back on track of being able to hear better very quickly’. (CI15) ‘Just… by sharing… with like‐minded handicaps of hearing and interesting journey. Sometimes discover somebody else with a cochlear and we exchange experiences and what's happening and how we're going’. (CI09) ‘And so I joined [Hearing Support Organisation] and attended lip reading classes once a week for five years and that again was a great help. … It's coping with your hearing loss, so it gives you tips on little bits of guidance. You know where to sit when you go to a restaurant, for example, how to ask people nicely to recognise your disability and to help you know, by the way, that they talk and respond to you, you know, not hiding behind their hands and things like this, which people often do’. (CI15)	Enabler
(2) Assistive device	‘I'm not very good with the mobile phone because of my hands. I tend to accidentally hang up and things like that. I'd much rather not use the mobile, but I'm very happy talking to people on speakerphone…’ (HA11)	‘One of the things that they said to do was to listen to some application… that was on an iPhone or something. And their ability to actually offer suggestions and assistance in the remediation or the rehabilitation process was pretty limited’. (CI14)	Barrier
	‘I do have all these different profiles on my phone. So, I've now got a profile that makes the television sound crystal clear. Only downside to that is when my wife speaks to me it's just horrible and it hurts’. (HA13)	‘They don't embrace the newest apps. They are hesitant in; they get very confused about how to use their technology… It slows down their progress at re‐engaging socially’. (CI08)	Both
		‘When I see my audiologist, if I tell her I'm having a problem with XY or Z, she'll tell me the best solutions or any new equipment that's available that might be of any benefit to me, and she'll organise it for me if I need it’. (CI02)	Enabler
**Theme 3: Patient self‐management**			
(1) Self‐advocacy	‘Because I've always said to them, I have trouble communicating with anybody because of my speech impediment. And it took a long time, you know, here I am for years and years, years. I thought I spoke clearly. I thought I spoke well. Obviously, I don't because a lot of people have said you don't talk properly’. (HA04) ‘But when I explained to kids that I'd just got hearing aids, they were really very good about. I explained that it was important to me that I didn't have more than one person speaking at the time and things like that. I think teenagers are terrific when they're given reasons and things like that’. (HA11)	‘I've had to push along the way, and I've had to become quite vocal to get further treatment. You get told one thing and then I've had to, like, reaffirm with them that I'm having big problems and I need it fixing. Because I wanted to go back to school, to study and so forth’. (CI16)	Enabler
**Theme 4: Accessibility to hearing services**			
(1) Funding	‘Try well accessing audiologists as I said we don't have audiology services… I live in [a town] and we have them at [government‐funded hearing clinic] and most people… are not eligible, for free service…’ (HA01)	‘so all of the questions that they have, which apparently were the only set of standard hearing impairment questions, were questions that would have been perfect if you were someone who was nonverbal and used AUSLAN. But for someone who's verbal, does not use AUSLAN, they had no relevance to me, so I had to answer never to all of these questions, which then made me look like I didn't had’. (CI05) ‘But it just seemed to add another level of complexity that took many months to get the report through NDIS before I got any approvals and by then the technology changed so much… But nine months later, …that's been superseded by a new model already, and so that's the situation I've found myself now is that I've had part funding of an aid that was recommended two models ago’. (CI06)	Barrier
	‘It was a free hearing thing in [Town]. And I went into it. And the person that tested me said yes, you do have hearing loss and I do think it would be helped by, by hearing aids’. (HA11)	‘So, my private health insurance covered the cost of the processor. Covered the cost of the operation and I suppose from here on Medicare is actually covering the cost of ongoing support through [hearing clinic]. If I did go through… a private ENT surgeon, I'm sure that the cost of me getting that ongoing support would be substantially more expensive’. (CI14) ‘This other audiologists who I'm with now, he was basically more interested in giving you a device and still is, in the device that is best suited to your hearing loss. And they've never been expensive, they've always been reasonably priced. So, I've never felt that I was being targeted to have to pay for you know, very expensive hearing devices’. (CI01)	Enabler
(2) Access		‘Yes, just once a year I have to do a map check. So, rather than going into the care centre I can do it remotely on my phone. So, I can go through all the sounds on my phone and the resource is collected and sent to the old audiology and they will tell me whether or not I need to come in or not’. (CI10)	Enabler
		‘I threw a dart at the board and came up with a person who was close… didn't know how to go about it, but there was … an audiologist just walking distance from where I worked’. (CI15) ‘We have [hearing centre], in [city], very close to me… And if they had spare parts for Cochlear and not just hearing aids, that would make life a lot easier too’. (CI10)	Barrier

#### 3.2.1 Theme 1: Hearing health‐care services

##### Primary health‐care services and specialists

General practitioners (GPs) were reported to be the primary medical contact to seek assistance with hearing issues. Both HA and CI users voiced mixed opinions regarding the clinical competency of GPs in managing hearing health. Some users expressed concerns about the GP's limited knowledge and poor communication in managing their hearing concerns, while others reported positive experiences, noting that their GPs were proactive in facilitating further medical evaluations through referrals to otolaryngologists.

##### Hearing services

Audiologists' knowledge and communication are crucial for the transfer and sharing of information. Some HA users reported that their clinicians were sales‐focused, and they had received unclear information about the hearing intervention process. Some participants reported their needs were not met after receiving their HAs due to the audiologist or audiometrist not adequately addressing their concerns, except for two HA users (HA05, HA09). Several CI users reported that their audiologist recommended a CI candidature evaluation when their current hearing devices or hearing acuity were inadequate for daily activities. One CI user (CI03), however, raised that their audiologist had not given enough consideration to their mental health requirements related to their hearing loss.

#### 3.2.2 Theme 2: Alternative support services

Support services were found to be crucial for individuals who have hearing loss. These services included third‐party support and device assistive services.

##### Social support and services

HA users found that receiving assistance from third‐party members, including family or members of their social club, had prompted them to seek medical attention for their hearing problems. Additionally, receiving support had markedly enhanced the likelihood of positive experiences with their hearing devices. Similarly, CI users reported that hearing support groups and spousal support had improved their postimplantation process.

##### Assistive devices

One HA user (HA13) expressed the importance of hearing services, especially text phone services, as these significantly impacted their daily activities. They suggested that phone call services could be improved to better cater to hearing impaired needs. One participant expressed mixed feelings about using hearing aid phone applications (apps). While the app altered the hearing aids' settings and enhanced their hearing acuity for activities like watching television, it made communication with their spouse challenging and uncomfortable. For CI users, an app was introduced for rehabilitation, but this was not found to benefit the users. Some CI users indicated issues concerning knowledge transfer from the audiologist around the usage of hearing technology, resulting in the lack of confidence to use their devices, particularly the non‐technology savvy users. However, one CI user (CI02) mentioned that with the support of the audiologist, their experience with the apps had improved.

#### 3.2.3 Theme 3: Patient self‐management

##### Self‐advocacy

Both groups mentioned the need to advocate for their own hearing loss at work or in personal matters. One HA user (HA11) shared that raising awareness about their hearing loss at work had improved workplace efficiency, while one CI user (CI16) shared that they had taken a proactive approach in initiating a hearing health‐care plan to advance their treatment.

#### 3.2.4 Theme 4: Accessibility to hearing services

Participants faced challenges with access and funding, which hindered them from timely CI and/or HA uptake.

##### Funding

Some CI users reported limited government funding and stringent eligibility had prevented them from promptly receiving a CI. One user (CI03) mentioned being on a waiting list to benefit from the government CI program. Hearing aid users also indicated stringent requirements that needed to be satisfied to benefit from government‐funded services. One participant (HA01) mentioned that it had brought about inconvenience in finding a hearing centre, as they did not meet the neighbouring clinic's requirement to benefit from the system.

For participants who were applying to the government fund relief, a questionnaire that gravitated towards individuals with severe deafness was provided and was not relevant to their situation (CI05). Another CI user (CI06) reported follow‐through issues between hearing centres and the Government National Disability and Insurance Scheme (NDIS) service centres. The NDIS is the Government welfare fund for citizens with significant disability and is available to individuals aged 65 years and younger.^14^

External factors had enabled both HA and CI users to access hearing health care. For one HA user (HA11), the provision of free hearing screening had propelled them to gain awareness of their hearing loss and progress onto the next step of hearing management. For CI users, one CI user (CI14) mentioned private health insurance had covered the cost of their operation, their device and ongoing rehabilitation, while another user (CI01) mentioned their audiologist had guided them through the process of ensuring the device was affordable for them.

##### Access

Some CI users reported that accessing remote support had led to significant improvements in postimplantation management. One CI user (CI10) mentioned attending telehealth appointments with the help of assistive technology. However, one user (CI15) reported difficulty finding information on or a location to get hearing management, particularly in rural areas. One CI user who utilises both HA and CIs mentioned the inconvenience of going to two separate clinics for hearing device maintenance (CI10).

## 4 DISCUSSION

This study explored barriers and enablers in Australia's hearing health‐care system for 31 adults aged 40 years and older, using HAs and/or CIs. Key themes that were identified included hearing care management, integrated supports, patient self‐management and accessibility.

### 4.1 Clinical processes

In the context of hearing loss management, primary health‐care providers play a critical role in early detection and referral for appropriate treatment.

General practitioners were often the first point of contact for participants when seeking help for their hearing loss. However, the lack of knowledge and/or confidence of GPs and audiologists prevented individuals from receiving optimal health‐care services. Our findings aligned with Bennett et al.'s^6^ study suggesting that a lack of knowledge or confidence among GPs can adversely impact their clinical judgement in addressing the patient's hearing loss and associated concerns. To improve the clinical management of hearing device users, recent guidelines developed by the CI Task Force,^15^ adapted for use in Australia, could be implemented for improved primary hearing screening and management. Primary health professionals could perform basic hearing screening, as adapted from Strawbridge et al. (2017), by asking patients if they have noticed any signs or symptoms of hearing loss.^16^ It is recommended that this question be initiated for patients aged 50 years and older.^16^ The updated Royal Australian College of General Practitioners (2024) guidelines for managing patients with hearing loss introduced the use of questionnaires, such as the Hearing Handicap Inventory – Screening Version (HHIE), while discontinuing the use of whispered voice and finger rub tests for screening.^17^ Additionally, the study's findings suggest the need to address the psychosocial aspects of individuals experiencing concerns related to hearing loss. Common signs of hearing loss and the consequences of delayed detection and intervention should be clearly explained to patients to provide them with the contextual knowledge to make informed decisions about their hearing health. Moreover, patients and health‐care professionals need a defined standard‐of‐care pathway to optimise the management of hearing loss in adults. General Practitioners could partner with practice nurses, practice managers, and local audiologists to enhance hearing loss detection and intervention rates.^6^

Secondly, participants reported that audiologists had insufficient knowledge on CI referral. Managing concerns around the pre‐ and postimplantation process and costs involved were identified as being important to participants. Similarly, these factors were highlighted in similar studies investigating barriers and facilitators to CI uptake,^10^ as limited knowledge of CIs, eligibility criteria and the referral process acted as barriers to CIs assessment referrals by health‐care professionals. A possible solution would be to introduce a screening tool for referral to aid in individualised counselling^18^ and having candidacy and referral guidelines for audiologists^19^ would build their confidence in supporting patients who might be eligible for a CI.

Thirdly, the study's findings highlighted the importance of postcare services that address the users' needs rather than depending on the functions of the HAs. One HA user reported that their audiologist struggled to address their psychosocial needs, while other users mentioned the lack of continuity of care, which affected their experience with the practitioners. This observation corresponded with findings from previous studies, which highlighted similar concerns among individuals with hearing impairment seeking hearing care treatment.^20^, ^21^ Recently, Timmer et al.^22^ proposed a five‐step framework to assist hearing care clinicians in addressing the social and emotional needs of adults with hearing loss. This framework emphasises the integration of family support and acknowledges the psychosocial challenges associated with hearing loss, advocating for a personalised approach to meet the unique needs of the client. Audiologists could consider modifying their communication during appointments to adequately address the needs of patients more holistically.^21^ Additionally, the use of validated questionnaires, such as the Hearing Acceptance Questionnaire, could help audiologists better understand the psychosocial needs of the patients.^23^ Effective clinical communication can be achieved through a clearer understanding of the patient's perspective and lived experience.^20^, ^21^

These findings reiterate the importance of tailored, evidence‐based approaches in optimising the health‐care pathways for adults with hearing loss, ensuring that they receive the comprehensive support they need to thrive in daily life.

### 4.2 Health education and patient engagement

Hearing aid users pointed out the misalignment between their expectations and the audiologist's or GP's approach in providing support. Furthermore, CI users reported not feeling confident to act on the information received from their audiologist to manage their hearing loss. It is important for individuals to take the initiative to educate themselves about hearing loss and its management, as self‐efficacy is positively linked to HA uptake and better rehabilitation outcomes.^8^ Gotowiec et al.^24^ proposed that empowerment on the hearing health journey requires knowledge, skills and strategies, self‐efficacy, participation and control of their hearing health and everyday lives. Audiologists play a role in providing adequate support to ensure that the patient is competent in using and managing their device.^8^

Additionally, there needs to be a focus on building trusting relationships between patients and health‐care professionals. To provide effective hearing care, professionals should adopt a person‐ and family‐centred care approach (PFCC); a holistic model that fosters balanced decision making between the individual with hearing loss and the clinician,^25^ while recognising the vital role of their family and support network in the hearing loss journey.^26^ This means considering the individual's demographics, such as age and gender, as well as their social influences,^25^ including the attitudes of family and friends.^26^ By adopting a person‐centred approach, professionals can better support their client's overall health and well‐being.^23^ This involves considering the individual's needs, educating them about their condition and referring them to appropriate services. It also includes reviewing communication strategies^20^, ^21^ and clinical competencies^21^ that impact patient care, as well as evaluating surgical workflows for cochlear implantations in hospitals. Addressing these challenges is essential to ensuring individuals with hearing loss receive the necessary support to navigate hearing health care effectively.

### 4.3 Integrated support

This study found the involvement of family, friends and other health‐care professionals to be a motivator for patients seeking help, while also acknowledging the significance of third‐party organisations or peer support in motivating individuals to address their hearing loss. It is important to recognise the significance of familys and friends' support,^26^ as well as assistive listening accessories^27^ which can greatly improve an individual's life. It is crucial for alternative rehabilitation and support services, such as support groups and primary hearing care services, to collaborate and create a seamless holistic hearing care experience for patients.^28^

For CI users, the support from family and clinicians, and the simplicity of using CIs, facilitated the postoperative experience; surgical complications, durability and cost of maintaining the device challenged this experience. Seeking assistance from established support groups could provide support during the postoperative phase.^28^

For HA users, low levels of digital literacy led to user frustration on usage and a sense of being left behind in a world that increasingly relies on technology. Previous research indicated that HA and hearing‐related smart‐phone apps helped individuals regain a sense of control over the challenges posed by their hearing loss.^29^ Selecting the appropriate support tools or apps is crucial for facilitating rehabilitation and support services, and enabling hearing device users to function effectively in their daily lives.^29^

### 4.4 Accessibility to health‐care services

Government funding has played a significant role in improving access to hearing services. While self‐support is essential in facilitating adaptation to hearing loss, it may not always suffice, particularly when government funding is limited and patients must rely on private insurance to fund their implantation. In Australia, funding models for adults aged 40 years and older include the NDIS^14^ and the Hearing Services program,^30^ which supports individuals under 26 years and older adults who are pensioners, veterans or concession cardholders. It is imperative to adjust the range of coverage provided by the Government based on the results of assessment. Although individuals with hearing loss can access the Government NDIS, administrative issues may impede access to the funds. Addressing and resolving these issues is essential to ensure that hearing services are delivered in a smooth, streamlined and cost‐effective manner.

Standardising pricing across all hearing centres is crucial to establishing equitable access to hearing services. Separating the device manufacturers and audiologists can help clients/patients feel more comfortable while receiving services or making the appropriate decision during their device purchase. To achieve this, policy makers should work together with professional audiology bodies to understand the business model better and regulate pricing across Australia. Providing transparency about hearing care operations and pricing will help build client/patient trust and confidence in the service‐model.^7^ Consumers will also be better informed about the structure and pricing of services and HA models, enabling them to make informed decisions.

Private insurance cover can help individuals access the services they need to improve their quality of life. However, it is essential to establish a rehabilitation plan that considers all available support services. Financial considerations around purchasing a CI or HA play a significant role in influencing a patient's decision to move forward in their hearing health‐care journey.^7^, ^9^ Hence, being transparent about costs during the initial consultation and providing multiple options would enable clients to feel confident in their decision making and lead to a smoother interaction with their audiologist.^7^

Factors related to accessibility, such as location and making easy‐to‐read educational materials available, must be considered to ensure seamless hearing health care for patients with hearing loss. While there was limited mention of tele‐audiology by our participants, with only one user commenting on its usage, it is acknowledged that tele‐audiology has grown in recent years, covering aspects of the patient journey, particularly the fitting and postfitting phases.^29^ Tele‐audiology holds promise in increasing cost‐efficiency, enabling better access to care and improving patient outcomes and satisfaction.^29^ However, further research is needed to increase the uptake and efficacy of tele‐audiology, especially around technical and clinical validation, and the optimisation of service delivery. Additionally, policymakers should consider providing guidance to individuals with hearing loss, including developing an appropriate website to help people with hearing impairment better understand the process of managing their hearing problems and navigating through the hearing health‐care system. This will enable them to make an informed decision based on reliable information. Additionally, aftercare support for CI recipients is sub‐optimal, particularly for those transitioning to bimodal use, as they must manage two different devices and spend additional resources and time on maintenance.

While our study displayed strengths, including its high participation rate and qualitative design, it also revealed limitations, notably limited representation from some Australian states, potentially limiting generalisability of study findings. Future research should integrate diverse cultural/ethnic backgrounds, location accessibility and device usage in their study design to fully capture the needs of the adult population with hearing loss.

## 5 CONCLUSIONS

This study highlighted the barriers and facilitators faced by HA and CI users, including clinician knowledge, psychosocial support, funding and accessibility to health care. Addressing these factors may help establish a holistic and transparent hearing health‐care service for all hearing device users.

## FUNDING INFORMATION

This work is supported by Macquarie University and Cochlear Ltd. Cochlear Ltd. has not played nor will play a role in the design, execution or reporting of the study.

## CONFLICT OF INTEREST STATEMENT

Jessica Turner and Bamini Gopinath reports financial support was provided by Macquarie University and Cochlear Ltd. There are no other conflicts of interest.

## Supporting information


Appendix S1

## Data Availability

The data that supports the findings of this study are available in the supplementary material of this article.
