# Supplementary material for: Understanding hearing health‐care access in Australia: Users' perspectives
Source: Australas J Ageing. 2025 Apr 22;44(2):e70029. doi: 10.1111/ajag.70029 (PMC12012597; doi:10.1111/ajag.70029)
Supplement: Supplementary file 1 — Appendix S1 [file AJAG-44-0-s001.docx]

**Appendix** **S1**

Interview questions exploring hearing journey among participants.

| *Hearing-related characteristics* |
| --- |
| 1. Could you describe the hearing loss that you have (e.g. does it affect one or both ears, how severe is the hearing loss, how long have you had a hearing loss, does it run in the family)? |
| *Questions regarding patient experience of hearing health services* |
| 1. Could you tell me about the treatment/s or services you have received or accessed specifically for your hearing loss (e.g. visiting GP/audiologist and/or ENT, use hearing aid/cochlear implant, duration of use, any other treatment/rehabilitation)? |
| 1. Could you describe the steps or the referral pathway you went through from finding out you have a hearing loss to receiving the treatment (hearing aid, cochlear implant and/or other) rehabilitation you needed? |
| 1. Following your treatment/rehabilitation (hearing aid fitting/cochlear implantation), have you continued to receive adequate support from your hearing health service provider or any other healthcare professional?   - Detail of any post-treatment support offered and accessed |
| 1. On a scale of 1 to 10 where 10 is very positive, how would you rate your experience throughout this hearing health service journey? Why did you give it this rating? |
| 1. Based on your experience, how could the current hearing health service be improved or modified? Could any changes be made to make the process easier or the experience better? |
| 1. Do you have any further comments regarding your experience or anything else we have spoken about today? |
